# Host Transcriptomics as a Tool to Identify Diagnostic and Mechanistic Immune Signatures of Tuberculosis

**DOI:** 10.3389/fimmu.2019.00221

**Published:** 2019-02-19

**Authors:** Julie G. Burel, Mariana Babor, Mikhail Pomaznoy, Cecilia S. Lindestam Arlehamn, Nabeela Khan, Alessandro Sette, Bjoern Peters

**Affiliations:** ^1^Department of Vaccine Discovery, La Jolla Institute for Immunology, La Jolla, CA, United States; ^2^Department of Medicine, University of California, San Diego, La Jolla, CA, United States

**Keywords:** transcriptomics, infectious diseases, tuberculosis, host immune response, human immunology

## Abstract

Tuberculosis (TB) is a major infectious disease worldwide, and is associated with several challenges for control and eradication. First, more accurate diagnostic tools that better represent the spectrum of infection states are required; in particular, identify the latent TB infected individuals with high risk of developing active TB. Second, we need to better understand, from a mechanistic point of view, why the immune system is unsuccessful in some cases for control and elimination of the pathogen. Host transcriptomics is a powerful approach to identify both diagnostic and mechanistic immune signatures of diseases. We have recently reported that optimal study design for these two purposes should be guided by different sets of criteria. Here, based on already published transcriptomics signatures of tuberculosis, we further develop these guidelines and identify additional factors to consider for obtaining diagnostic vs. mechanistic signatures in terms of cohorts, samples, data generation and analysis. Diagnostic studies should aim to identify small disease signatures with high discriminatory power across all affected populations, and against similar pathologies to TB. Specific focus should be made on improving the diagnosis of infected individuals at risk of developing active disease. Conversely, mechanistic studies should focus on tissues biopsies, immune relevant cell subsets, state of the art transcriptomic techniques and bioinformatics tools to understand the biological meaning of identified gene signatures that could facilitate therapeutic interventions. Finally, investigators should ensure their data are made publicly available along with complete annotations to facilitate metadata and cross-study analyses.

## Introduction

Tuberculosis (TB) is a major infectious disease affecting one-third of the world's population. Infection with *Mycobacterium tuberculosis* (*Mtb*) manifests as a spectrum of disease states ranging from asymptomatic subclinical infection (latent infection, LTBI) to active disease (ATB). Most latently infected subjects have a persistent immune response to *Mtb*-specific antigens but do not show any pathology, and can be considered healthy. Diagnostic tests for latent infection include the tuberculin skin test and interferon-gamma release assays, both of which essentially detect an immune response against *Mtb*. However, positivity to these tests only reflect past *Mtb* immune exposure, and thus cannot discriminate between individuals with eliminated or controlled infection from those with subclinical active disease. Throughout their lifetime about 10% of individuals with latent infection will progress to active disease which has significant morbidity and mortality. Diagnostic tests for active infection include direct detection of *Mtb* organisms in sputum, by smear and/or culture or by nucleic acid amplification (GeneXpert), and detection of abnormalities consistent with TB by chest X-ray. In some cases where microbiological tests are negative, the sole presence of disease symptoms such as chest pain, coughing up blood or breathing difficulty can be sufficient for diagnosis, particularly in areas with a high TB burden. Treatment of ATB requires prolonged aggressive antibiotic regimen, typically a combination of a minimum of four drugs over a 6-months period (1). It is also associated with severe side effects, especially in immune compromised patients, a high inter-individual variability in terms of effective dosing and successful drug combinations, and a high failure rate, due to inefficient patient follow up and the rise of drug resistant strains (1–3). Overall, there is still a limited understanding of pharmacokinetics and mechanism of action of anti-TB drugs, as well as their relationship with disease phenotype and individual genetic background.

Several challenges in the field of TB are currently being investigated that would address significant public health concerns: (1) to develop diagnostics that can more accurately reflect the spectrum of *Mtb* infection states, in particular identify individuals at risk of developing active disease in order to treat them prior to developing ATB. This would prevent transmissions and could ultimately eliminate the reservoir of infections. Whereas, Isoniazid (INH) preventative therapy of individuals with LTBI is currently the primary prophylaxis for preventing TB progression, given the costs and side effects of treatment and the low rate of progression to active disease, it would be highly beneficial for both social and economic reasons to identify the individuals that will truly benefit from it beforehand. (2) Improving the specificity and sensitivity of diagnostics for active disease. Some ATB patients are reverting to a negative IFN-γ release assay (IGRA) test or their sputum is negative for *Mtb* bacilli detection either by culture or GeneXpert. Other confounding factors for diagnosis of ATB includes the ubiquitous presence of non-tuberculous mycobacteria (NTMs) in the environment that present significant immune cross-reactivity with *Mtb* (4). However, both GeneXpert and IGRA can discriminate between *Mtb* and most NTMs, since they, in the case of IGRA, lack the antigens included in the test (ESAT-6 and CFP10). Chest X-rays of lung granuloma forming diseases such as sarcoidosis or aspergilloma often look very similar to TB, and in the case of sarcoidosis, immune cross-reactivity to *Mtb* antigens can also be detected in both blood and bronchoalveolar lavage (BAL) (5–7). (3) Identify novel targets for therapeutic interventions. The exact mechanisms driving inter-individual variability in the pathology and control of TB are still poorly understood. Progression to active disease is increased in *Mtb* infected individuals with immune suppression, such as anti-TNFα treatment or HIV co-infection (8–11), or other immune regulators used for transplantation (12). Risk factors for progression also include comorbidities such as for example type 2 diabetes (13, 14). Conversely, obesity was associated with lower active TB risk (15–17). Better characterization of these immune and metabolic dysregulations and their impact on TB disease progression could provide novel targets for therapeutic intervention designed for different subsets of patients. Thus, some of the main needs for TB control are the improvement of current diagnostic tools to better discriminate between the different TB disease states and a better mechanistic understanding of successful immune responses to control and eliminate the pathogen to develop novel therapeutic interventions.

Host transcriptomics in TB (and other diseases) is a powerful approach for the discovery of immune signatures. Transcriptomic analyses of human samples provide insights into which genes are expressed in the sampled tissue. By comparing such transcriptomic profiles in samples from subjects with a disease vs. those without, it is possible to identify genes that differ in their expression between the groups, and thus are part of a disease signature. Identifying such signatures has two primary benefits: First, transcriptional signatures can be used as diagnostic tools to identify affected individuals. Second, they can be used as indicators of which genes, pathways and cells are affected by a disease, thereby improving the mechanistic understanding of the disease and enabling the design of new therapeutic or prophylactic interventions.

**Figure 1:**
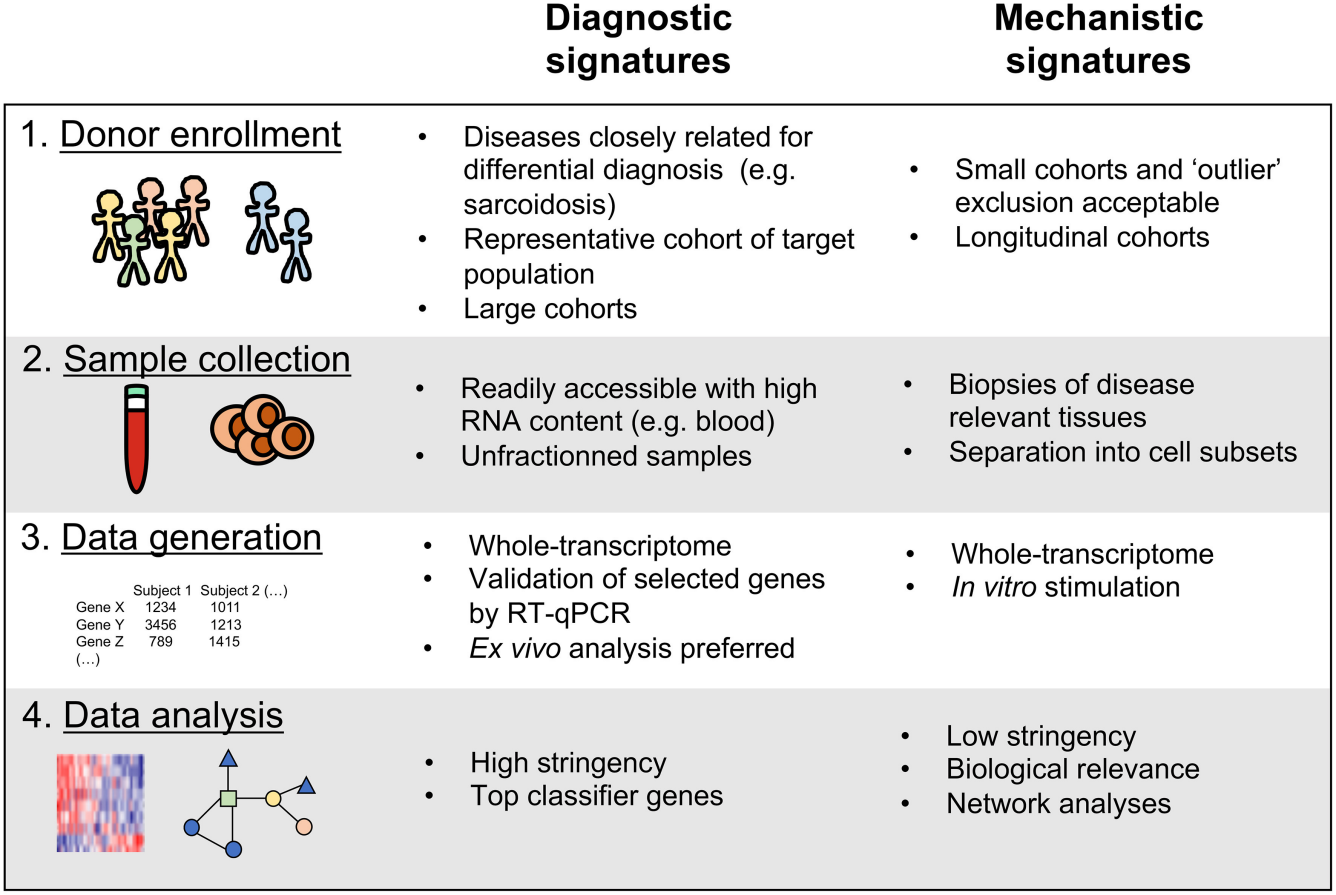
Key variables to consider for the discovery of diagnostic vs. mechanistic transcriptional signatures of disease.

Regardless of their purpose, all host transcriptomics studies of human diseases share these key steps: (1) Enrolling study cohorts of patients in defined disease states and comparative controls, (2) Obtaining samples from the enrolled subjects, (3) Performing assays to determine the transcriptional profile in these samples, and (4) Analyzing the generated data to identify disease associated transcriptional signatures (Figure 1). We have recently reported that actual implementation of these steps in a given study can significantly impact the utility of the generated signatures as either diagnostic tools or mechanistic indicators (18). To gain insights into disease mechanisms it is important to determine, for example, how changes in gene expression affect biological processes and which cell types are responsible for the observed changes. On the other hand, for diagnostic purposes, the transcriptomics signature must be very specific for the studied disease state while the mechanism of action is less important. In addition, the clinical implementations such as ease of sampling should be straightforward. In this review, based on already published transcriptional studies of *Mtb* infection and our recently published commentary (18), we further develop these guidelines and envision how future studies can utilize the transcriptomic tool kit for both better diagnostic and mechanistic understanding of TB (Figure 1).

## Step 1: Study Cohorts

### General Considerations

Key variables to consider when enrolling cohorts of patients to discover transcriptomic signatures of disease are: (1) TB disease and infection states targeted and how they are defined upon enrollment; (2) Co-factors, such as presence of co-infection with HIV that could either be an exclusion criteria or part of the clinical variables captured; and (3) Cohort study design, encompassing cohort structure, number and size, and cross-sectional vs. prospective cohorts.

In terms of disease states targeted, the most simplistic study should consider the following three categories: uninfected subjects (NoTB; who have or have not received BCG vaccination depending on the location of the study), healthy subjects with latent infection (LTBI), and subjects with active disease (ATB). The NoTB group serves as a comparator to identify a TB disease-specific signature. Subjects in this group are thus enrolled based on diagnostic criteria that rule out TB infection. The stringency of these criteria can be adjusted based on the likelihood of having false negative results. An additional control group can consist of individuals with diseases that have similar symptoms as TB, but require a differential diagnosis, such as sarcoidosis or other lung pathologies. As for the TB infected cohorts, TB is known to manifest as a spectrum of disease and infection states, so neither the LTBI or the ATB group should be considered as a class of homogeneous individuals (19). For instance, LTBI subjects can be divided into low and high responders based on the magnitude of their IFNγ response to *Mtb* antigens (20). LTBI subjects with high IFNγ responses clustered closer to active TB individuals, suggesting their LTBI status might actually represent subclinical active disease (20). Knowledge regarding co-morbidities may also help determining individuals that are most likely to progress to ATB (21). For ATB, subjects can be divided based on their diagnostic test results, not only to rule out false positives, but also to get an idea of their disease severity (22), or by distinguishing between pulmonary and extra-pulmonary TB (23, 24). Further segregating the active disease cohort will inform physicians on the best treatment regimen to adopt for each patient or immunologists on the mechanisms driving TB dissemination. Classification based on drug resistance will enable physicians to effectively choose second or third-line therapies. Thus, current diagnostic tests could benefit from extra segregation within the LTBI or ATB state. Alternatively, the development of one universal test that could distinguish all possible infection states across the TB disease spectrum would greatly improve our current definition of patient cohorts and treatment options.

In terms of co-factors, in general it is important to get as much information as possible on subject's adverse health status that could impact host transcriptomic signatures, such as comorbidities, other infections, demographics and geographic location. Specific attention should be paid to pathologies or infections that can be highly prevalent where subjects are being recruited. For instance, type 2 diabetes, which is a known risk factor for developing TB and has a growing incidence in TB endemic areas (25), was shown to significantly impact TB associated blood molecular signatures in co-affected patients (26–28). Information about prior immune status should also be reported, since seropositivity to many common viruses such as herpes virus or CMV might also affect blood transcriptomic signatures (29) and ATB risk was associated with increased responses to CMV (30). Demographics are also an important factor to take into consideration: children are associated with distinct TB transcriptomic signatures compared to adults (22, 31). Gender and ethnicity are known to interfere with host immunity and susceptibility to TB infection (32, 33), but have not been studied yet in the context of transcriptomics. Additionally, geographic location of the study cohort is a key parameter that needs to be considered. Diverse geographical locations are associated with exposure to distinct microorganisms, which might impact how the host will react in response to a given perturbation. There is increasing evidence that microbiome can also shape TB-specific immunity. Our recent work indicated differential epitope responses as a function of TB disease history, with a specific set of peptides whose reactivity is lost early after treatment. These peptides show high conservancy across NTMs and other commensal bacteria, and are thus likely targeted by anti-TB drugs in a non-specific fashion (34). In a mouse model of infection, commensal microbiota was shown to have a protective effect on lung colonization by *Mtb* that was partially mediated by mucosal-associated invariant T (MAIT) cells (35).

Finally, a key variable to consider is the cohort study design (i.e., structure and size), which is dependent from all previous variables described in this section. Clearly defining the study objective from the start will dictate which disease populations should be studied, the co-factors to be considered and the appropriate controls to be included. In terms of structure, some studies contain only one cohort, while others divide their subjects into training, test and validation cohorts. The training set is used to build the transcriptional signature, which is then verified in the test cohort and sometimes in a third independent validation cohort. The number of subjects within each cohort is highly variable, ranging from a few samples to hundreds of samples. The number of cohorts and cohort size depends on human subject and sample availability, and on the study objective, diagnostic vs. mechanistic, as described below. The majority of studies are cross-sectional, but prospective studies including longitudinal cohorts can be valuable for instance for studies of treatment efficacy (36–38) or for progression from latent to active disease (39–41).

### Diagnostic Studies

For diagnostic studies, it is crucial to take into consideration diseases closely related to TB in a separate cohort to ensure the specificity of the gene signatures identified. A major breakthrough in TB-related transcriptional studies came with the identification of an interferon driven gene-signature from whole blood samples that was able to distinguish subjects with ATB from NoTB or LTBI (42). However, a follow up work showed that this interferon signature is not specific to ATB since patients with sarcoidosis, a lung homing disease that also develop granulomas, showed that signature as well (43). Additionally, the major co-factor that can confound the efficiency of diagnostic signatures are co-infecting diseases that are highly prevalent in TB endemic areas. Indeed, the transcriptomic signature used to diagnose active TB obtained from HIV seronegative individuals cannot be as efficiently reproduced in HIV seropositive subjects (44, 45). Thus, transcriptomics studies that aim to design novel TB diagnosis tests to be used in the clinic should include cohorts with and without important co-factors such as HIV infection or sarcoidosis to ensure the robustness of newly identified signatures of TB.

In terms of cohort size, transcriptomic studies for diagnostic purpose should aim to get large numbers of samples to reach adequate statistical power and robustness. Since such studies aim toward highly reliable and reproducible signatures “the more—the better” rule fully applies here, and appropriate power calculations must be made to assess the minimum number of samples to be included in each cohort. Most successful studies for the identification of transcriptomic signatures of TB for diagnostic purposes were based on cohorts of hundreds of cases (22, 42, 44).

Finally, for diagnostic studies, it is crucial to validate any signature in a study population that mimics the actual patient population for which the diagnostic would be applied. While it is possible to define a diagnostic signature in cohorts that exclude patients with co-morbidities or less clearly defined disease states in order to identify a stringent disease signature, the diagnostic utility of such a signature can only be assessed in a general patient population. Thus, it is especially important to include subjects from another ethnicity or geographic location as a validation cohort to test the robustness of the transcriptomic signature. For instance, Berry et al used an initial training/test set from UK, and then used a validation cohort from South Africa (42). Diversifying the geographic location of disease cohorts can also be important to rule out the influence of disease transmission rate in host transcriptomic signatures. For instance, most work in blood TB transcriptomics has been done in South Africa which has one of the highest TB transmission rates worldwide, and could explain the lower specificity and sensitivity of these signatures to classify cohorts from regions with low TB endemicity, such as the US (46). Indeed, variations in *Mtb* exposure rate, BCG vaccination, and circulating NTM strains will influence TB-associated immune responses, and thus host transcriptional signatures in areas that are TB endemic vs. low transmission areas.

### Mechanistic Studies

While a diagnostic signature should be present in every individual, a mechanistic signature might not necessarily be shared by the entire cohort. Indeed, as aforementioned, TB is associated with high inter-individual variability in terms of disease symptoms, diagnostic test results and immune response patterns. Thus, one might actually gain in information by dividing cohorts into refined subgroups. Cohort subdivision can be done based on known clinical or immune parameters (e.g., pulmonary vs. extra-pulmonary for ATB, progressors vs. non-progressors in the case of LTBI) or in an unbiased fashion based on data clustering tools.

Another important consideration in mechanistic studies is to differentiate between primary and secondary effects (i.e., cause vs. consequence) within transcriptional signatures. Although cause and effect in biological processes (including pathogenesis) are typically interconnected in circular patterns, it can still be informative to define whether a dysregulated gene expression is the direct result of infection (e.g., IFNγ production by antigen-specific T cells) or the consequence of the primary response (recruitment of neutrophils and monocytes to the site of infection). To tease apart these two effects can be challenging, especially on cross-sectional studies of mixed cell populations, as described in the sections below. A way to address this issue is to obtain longitudinal samples that can account for the kinetics of the response to infection and help separate cause from effect. For example, the recent study by Scriba et al. delineates the kinetic events occurring in the blood transcriptome of healthy LTBI subjects before progression to active disease (47). By analyzing the blood transcriptome at repeated times until progression, the authors were able to develop a hierarchy within overall biological dysregulations detected, with changes in type I/II IFN modules preceding any other changes, followed by monocyte and myeloid inflammation modules and finally lymphocyte modules (47).

## Step 2: Sample Collection

### General Considerations

Two main parameters should be considered for sample collection in transcriptomic studies: (1) the nature of sample collected (i.e., tissue or bodily fluid) and (2) whether the sample will be used as a whole or separated into cell subsets.

In terms of tissue or bodily fluid, the most readily accessible samples from humans are blood and urine. Urine is a non-invasive sample to obtain, but contains very little RNA information. Conversely, blood collection requires a generally well-tolerated invasive procedure but contains a high diversity of cell populations. Because of the constant circulation of cells between blood and organs, blood-based studies can also give information on the global health status of a given individual at the time of collection. The main drawback of accessing cells from the peripheral circulation is that they are not reflecting what is happening at the site of infection. In contrast, biopsies of infected tissues, and/or draining lymph nodes can give precious information regarding pathology and antigen-specific cells (48, 49). In the case of lung manifested infections such as TB, bronchoalveolar lavage (BAL) or sputum can be obtained from a relatively non-invasive procedure and contain information on immune cells present at the site of infection. As an example, Garcia et al found both *Mtb* and host immune related genes dysregulated in sputum and BAL of ATB patients (50).

The initial sample collected is often further processed, resulting in the removal or enrichment of specific cell populations. For instance, blood samples are often processed to peripheral blood mononuclear cells (PBMC), because PBMC can be readily frozen and stored for subsequent analysis, while whole blood has to be stimulated and/or stained for flow cytometry immediately. PBMC samples no longer contain granulocytes (neutrophils, eosinophils and basophils) and platelets, which can contribute to the disease signature. For instance, the whole blood IFN-inducible gene signature reported by Berry et al. was dominated by neutrophils (42), which represents 50–70% of white blood cells in humans (51). While using whole blood might thus seem advantageous because it contains more cell types, at the same time, it also means that transcriptional signatures from less frequent cell types will be “drowned out” by the more copious RNA from abundant cell types. Thus, in some cases, it might be more informative to break down samples and generate transcriptomic signatures of isolated cell subsets of interest. For instance, while it is not possible to differentiate between LTBI and NoTB subjects using whole blood (42, 52), differentially expressed protein- and non-protein-coding RNAs were apparent in CD4 T cells derived from the two subject cohorts (53, 54). Cell subsets can be isolated by fluorescence activated cell sorting (FACS) or by negative or positive selection using magnetic based sorting. FACS yields cell populations with high purity and offers the possibility to use multiple combinations of surface markers. However, it is a more complex procedure compared to magnetic negative isolation that keeps the cell populations of interest and thus their RNA content “untouched.”

### Diagnostic Studies

In diagnostic studies, sample availability is a critical factor to consider. The ideal diagnostic test should be based on a small volume (i.e., <5 ml of blood sample volume or <5 × 10^6^ cells) of a biological sample that is readily accessible in any human subpopulation (e.g., sick individuals and any age-group; infants to elderly). Thus, blood is a good sample type for diagnostic transcriptomics, as it is readily accessible and contains a high RNA and cellular concentration. Several blood-based transcriptomic studies have been undertaken to improve TB diagnosis. As mentioned above, the seminal report by Berry et al. identified a 393-gene signature able to discriminate between ATB vs. LTBI (42), and was followed by many others. To name a few, these studies have led to the discovery of gene signatures that can discriminate ATB from other lung diseases (43, 55), as well as gene signatures that can differentiate between ATB vs. LTBI in HIV seropositive adults (44), and in children (22).

Additionally, a good diagnostic test necessitates a robust workflow that requires minimal resources to ensure feasibility and reproducibility in the highly variable clinical infrastructures worldwide. The fewer sample processing steps that are needed, the easier it is to ensure consistency between sites. Thus ideally, unfractionated samples, such as whole blood should be used and FACS sorting or other complicating cell separation techniques should be avoided.

### Mechanistic Studies

Unlike diagnostic studies that aim for the most readily accessible samples and the least processing steps, for mechanistic studies, access to biopsies of disease relevant tissues and cell types in healthy and diseased individuals would be of highest value. However, obtaining such samples can be associated with complex ethical and practical hurdles, and will generally only be possible for a limited number of subjects. Although associated with reduced statistical power, studies with limited samples from disease relevant tissues can still have high informative value. For instance, microarray analysis on a handful of lung biopsies from TB patients was sufficient to highlight immune and inflammatory functions/pathways commonly dysregulated in TB granulomas (48, 56). Furthermore, these data showed little overlap with previously published whole blood gene signatures (48), implying that lung specific immune responses might be poorly represented in the peripheral blood. In another study, transcriptomics on infected lymph nodes from 13 extra pulmonary TB patients compared to uninfected lymph nodes identified distinct gene expression signatures from granulomas, enriched in genes involved in Th1/Th2 responses, as well as IFN-inducible genes (49).

Mixed cell populations samples such as whole blood can still be informative, but additional work should be performed in order to assign “cell responsibility” for genes in the observed signature. Minimally, investigators should use single-cell protein analysis techniques such as flow cytometry or CyTOF to determine cell sample composition and correct for this in data analysis, for instance with the csSAM algorithm (57). A more thorough approach is to do vast amounts of single cell sequencing, to account for both variability in cell subset composition and cell to cell variation within a given cell subset. A cheaper (and less bioinformatics intensive) alternative is to sequence isolated cell subsets to provide more focus on specific cell types that are likely involved in the control (or lack thereof) of the infection. For instance, transcriptomic analysis of memory CD4 T cells identified novel immune signature of *Mtb* exposure in the context of LTBI, and helped identified novel markers of *Mtb*-specific T cells (54). *Mtb*-specific T cells can also be isolated and further analyzed using tetramers with specific peptide/MHC combinations (58).

## Step 3: Data Generation

### General Considerations

The key two parameters to consider for data generation in transcriptomic studies are (1) the assay platform and (2) whether the sample will be stimulated or not.

The different gene expression platforms available have been discussed in detail elsewhere (59–61), and will thus be only touched upon briefly here. There are two major methodological approaches for the generation of transcriptomic data: (i) whole-transcriptome analysis and ii) targeted gene expression analysis. Microarray was the traditional method for whole-transcriptome analysis, but as the costs of sequencing methods are decreasing, there has been a transition to high-throughput RNA sequencing (RNAseq). RNASeq has several benefits over microarrays such as: (i) not requiring probes and thus it can detect in an unbiased fashion novel transcripts and expression of non-coding RNAs (which are now acknowledged as being key contributors to immune responses); (ii) a lower background signal because the cDNA sequences can be mapped without ambiguity to unique regions of the genome; (iii) a broader dynamic range to quantify gene expression levels; and (iv) more flexibility in meta-analysis, enabling the simultaneous analysis of different datasets (62). In contrast, targeted gene studies measure a handful of genes selected either from preliminary data, or belonging to a specific category (e.g., pathway, function, cell subset). In this case, data is typically generated by RT-qPCR, or as recently adapted in several studies, by RT-MLPA (reverse transcriptase multiplex ligation-dependent probe amplification) (39, 63). An emerging platform is the Nanostring technology (Nanostring Technologies, WA, USA) that uses barcoded mRNA-specific hybridization probes combined in custom or pre-made panels of up to 800 genes (64). This technique can be performed directly on cell lysates and allow for direct absolute quantification, and therefore represent an attractive intermediate approach between whole-transcriptome and targeted gene expression analysis.

In terms of *in vitro* stimulation, transcriptomic signatures can be derived from samples in different states of activation: directly *ex vivo*, or after global or antigen-specific *in vitro* stimulation. Transcriptomic signatures derived from unstimulated samples reflect the *ex vivo* state of the host. On the other hand, antigen-specific stimulation can give additional information on the transcriptional changes that could be occurring *in vivo* after encounter with the pathogen. Thus, the comparison of unstimulated vs. stimulated blood transcriptomic signatures will provide a pathogen specific readout that is more likely to reflect the disease specific immune response as opposed to blood signatures directly extracted *ex vivo* from a subject. The choice of the stimuli depends on the cell subset of interest: peptide pools will specifically activate classical CD4 and CD8 T cells, whereas lipid antigens will target non-classical T cells, and heat-killed *Mtb* or lysate will induce more global immune responses by activating a plethora of cell types. Live *Mtb* preparations are useful to study the transcriptome of infected cells such as monocytes.

### Diagnostic Studies

Ideally, a diagnostic test should be simple (e.g., PCR detection of a small number of genes) to ensure reproducibility. Moreover, a cheap test will enable a broader use, such as in low resource settings. However, deciding which genes should be included in a diagnostic test benefits from broad investigation. The most commonly used approach is to first perform whole-transcriptome screens to select the best discriminatory genes between cohorts of interest, which will be then validated at the individual gene level (23, 31, 41).

In an attempt to reduce logistics associated with a diagnostic test, direct *ex vivo* analysis (unstimulated samples) is largely preferred. However, antigen-specific stimulation can be considered in some cases, in particular to discriminate between similar pathologies. For instance, as mentioned above, it has been proven very difficult to differentiate between sarcoidosis and active TB when solely analyzing the *ex vivo* whole blood signature (43). Stimulation with *Mtb* antigens will specifically activate cells that are responsible for combating *Mtb*, making the detected transcriptional signature more specific, and removing potential convoluting signals (e.g., co-infections, non-disease specific inflammatory processes). Additionally, antigen-specific stimulation might be advised for diseases which have a too “weak” *ex vivo* signature [e.g., transcriptomic analysis of unstimulated whole blood/PBMC could not discriminate between LTBI and NoTB individuals (42, 52) whereas *ex vivo* isolated memory CD4 T cells could (54)]. Because antigen-specific stimulation can enhance disease specific gene expression compared to unstimulated samples, it increases statistical power to accurately discriminate between infected and uninfected samples. Successful examples of the application of *in vitro* stimulation in TB transcriptomics for diagnostic purposes include the discovery of gene signatures in blood after 6-day *in vitro* culture with live *Mtb* that can predict relapse after treatment of ATB (38) and candidate genes in PPD-stimulated PBMC that can distinguish latent TB from ATB (65).

### Mechanistic Studies

For mechanistic studies whole-transcriptome analysis is definitely the preferred approach for data generation because limiting analyses to individual gene transcriptomic assessment would typically not be justified. Additionally, since mechanistic studies are not restricted by costs associated with data generation, the use of more sophisticated techniques or emerging platforms is encouraged to increase the likelihood of discovering novel disease signatures. As an example, single-cell RNA sequencing is a promising emerging technique in the field of transcriptomics that can be used to explore cell to cell heterogeneity within a population, help re-defining cell subset classifications and reveal novel signatures of disease [reviewed in (66)]. For mechanistic studies focused on T cells, single cell TCR sequencing can be used to track antigen-specific T cells, and study the association between TCR repertoires, HLA type, and epitopes. A recent study showed the ability to identify CMV-specific TCR sequences based on blood immunoprofiling of 666 subjects with known CMV status (67). These sequences could then be used to infer the CMV exposure history of individuals and partially predict their HLA specificity. In the case of TB, TCR sequencing of TB-specific T cells identified distinct groups of TCR sequences that share *Mtb* antigen specificity (68). Moreover, TCR sequences can be used as a natural “barcode” which can be used to track cell origin. As single cell sequencing allows to obtain TCR sequence and transcriptome simultaneously it opens novel opportunities for profiling TB-specific cells at unprecedented resolution.

Antigen-stimulation can also present lots of advantages over unstimulated samples for mechanistic studies, in particular for T cells. T cells specific for a given antigen typically represent only a small fraction of circulating cells; hence their transcriptomic profile will be masked when investigating the whole blood transcriptome. One way to overcome this issue is to perform antigen-specific *in vitro* cultures in order to expand the cells of interest and their associated transcriptomic signal. For instance, whole blood from ATB patients *in vitro* cultured with live *Mtb* for 6 days identified gene signatures that can predict post-treatment relapse (38). Similarly, stimulation of PBMC from NoTB subjects with live *Mtb* has helped to define novel markers of antigen-specific T cells (69). Antigen-specific stimulation can also be combined with cell sorting to isolate antigen-specific cells. In the case of TB, IFNγ producing T cells could be successfully identified using either *Mtb* lysate (70) or comprehensive peptide pools (71). Mechanistic interpretation of transcriptomic data after *in vitro* stimulation should be done carefully by acknowledging the following two caveats: (i) a lack of response can be due to either non-responsive cells or migration of responsive cells from the compartment interrogated to the site of disease; (ii) *in vitro* stimulation only unravel the stimuli potential of specific cell types, but might not reflect what is happening *in vivo*.

## Step 4: Data Analysis

### General Considerations

The data analysis workflow is quite different for studies intending to discover diagnostic vs. mechanistic transcriptional signatures of disease. The one shared component is a set of initial algorithms to identify gene sets associated with different disease states.

The two main approaches to identify disease associated gene sets are: differential gene expression analysis and modular analysis. Differential expression analysis is a supervised method aimed at identifying the genes that are expressed significantly higher or lower in individuals having a certain condition in comparison to a control cohort. A differential expression analysis calculates for each gene the fold change in expression between two pre-defined groups of subjects (e.g., NoTB vs. ATB) and the associated *p*-value and multiple testing-corrected *p*-value as a measure of statistical significance. Typically, genes with corrected *p*-value lower than 0.05 are considered as differentially expressed. Stringency can also be increased by adding a cut-off in the fold change in expression to select most prominent genes. The DESeq package (available through Bioconductor) (72) is one of the most popular algorithms used for differential expression analysis, as it can be adapted to datasets with minimal number of biological replicates. In the case of large numbers of subjects in each cohort (or disease states), the identification of differentially expressed genes (DEGs) can be done using more conservative and general approaches such as for instance, the Student's *t*-test.

Modular analysis is an unsupervised method aimed at identifying clusters of genes that share a similar expression profile. The output is a list of gene modules of various sizes, with each gene only contained within one module. The expression profile of each module can then be compared between disease cohorts. Modular analysis is statistically more powerful compared to differential expression analysis as it partitions the dataset into discrete modules and thus decreases its dimensionality. Furthermore, modular analysis allows to evaluate if a given gene module is associated with a clinical or biological variable of interest (e.g., disease severity, frequency of a certain cell type, plasma cytokine concentration). This is done by considering the degree of correlation between the principal component of a module and the variable of interest. One of the most used modular analysis algorithms is the Weighted Correlation Network Analysis (WGCNA) (73). Each module that shows significant association in its expression between the different disease cohorts can be considered a separate transcriptional signature of disease.

### Diagnostic Studies

Diagnostic studies aim to identify the smallest possible set of genes that could serve to discriminate between different cohorts. Thus, the preferred approach for diagnostic gene selection is differential gene expression analysis, with stringent fold change and low *p*-value cut-offs to identify genes with the highest discriminatory power. Several strategies based on machine learning tools can then be applied to further narrow down and predict a concise set of top classifier genes. Most popular machine learning tools include random forest algorithms, which generates an ensemble of decision trees, support vector machine models and neural networks. The minimal gene set identified can then be used in clinical settings for disease assessment. Examples of promising potential diagnostic tools for discriminating between ATB and LTBI include a 4-gene signature (74), a 3-gene signature (75), and BATF2 gene expression (23) in whole blood. In terms of TB prognostic, a 16-gene whole blood signature was reported as a good predictor of ATB risk in LTBI individuals, and could be detected as early as 12 months before disease development (41). Recent efforts have reduced this 16-gene signature to a smaller set of 4 genes to efficiently predict progression to tuberculosis in multiple cohorts across various geographic location in Africa (76).

An alternative approach to top gene classifiers is to measure a so-called disease risk score (DRS). In its simplest form the sum of the expression of upregulated genes is calculated and then subtracted by the sum of the expression of downregulated genes. By imposing a certain threshold on DRS value all samples can be classified to a certain disease group. The main advantage of this method is that it is easier to use in resource-limited settings, since less advanced bioinformatic analysis is required. With certain mathematical elaborations, such approach has been also termed as “molecular distance to health” (42) or “molecular degree of perturbation” (27). The DRS score has been applied to assess response to TB treatment, where its magnitude was shown to reverse to the level of NoTB controls following successful treatment (36). One can also imagine the use of a DRS score as a discriminative parameter for disease risk (e.g., progression of LTBI to ATB). A common approach to search for genes suitable for DRS calculation is to employ the elastic net algorithm. This method was applied by Kaforou et al. to identify a signature of ATB (44). The authors came up with a 27-transcript signature distinguishing ATB from LTBI and a 44-transcript signature distinguishing ATB from other diseases. The same algorithm was implemented in a pediatric study by Anderson et al. where the authors identified a 51-gene signature in whole blood that could discriminate ATB from other diseases in children (22). Importantly, all these signatures were not significantly distorted by HIV infection status (22, 44).

Biological relevance of the genes selected in the case of diagnostics research is secondary. In fact, in many studies the resulting list of genes is often not easily, if at all, interpreted biologically [e.g., see (31, 45, 74, 77)]. Indeed, from the whole plethora of initially identified DEGs (usually comprising thousands of genes) only those that are robust and uniform will be selected, which are not necessarily the most biologically relevant. Minimal discriminating gene sets will likely contain genes from unrelated pathways, because genes with shared function would belong to a single module of co-expression. Effective diagnostics on the contrary requires genes of unrelated expression pattern allowing for maximum information gained per gene added. As a result, best candidate genes for diagnostics are not necessarily related, and unlikely to carry similar biological information.

Finally, the use of previously published data to perform comparisons and cross-validations is extremely important for diagnostic studies aiming for high reproducibility and robustness. It provides a way to evaluate the efficiency of newly identified signatures in more diverse cohorts, for instance with different ethnicity or geographic origin. For instance, Roe et al identified BATF2 has a potential biomarker to diagnose TB and confirmed its diagnostic value amongst different ethnicity groups (23).

### Mechanistic Studies

The cornerstone of mechanistic studies is biological interpretation of the obtained disease signature. This includes unraveling underlying molecular mechanisms and inferring their causal relationships. Knowledge of these relationships in turn can guide the future development of therapeutics that could intervene with upstream molecular targets.

In terms of identifying disease signatures, differential gene expression analysis can be performed in less stringent fashion compared to diagnostic studies, because even slight changes in expression of regulatory genes can be important markers of cell's transcriptional program. Modular analysis can be appropriate to investigate co-expression patterns and to identify gene clusters with shared regulation. As an example, modular analysis was successfully applied by Montoya et al. to gene expression data of *in vitro* differentiated macrophages under different conditions (78). The authors found that M1-type macrophages, known to be involved in TB immunity, upregulated a specific cluster of genes in response to IL-15 treatment, and identified IL-32 as a hub gene regulating that cluster. Further analysis revealed that IL-32 could induce antibacterial properties in macrophages, and that the IL-32 gene cluster was consistently upregulated in LTBI compared to ATB subjects across many datasets and cohorts. Thus, solely using modular analysis, the authors were able to uncover the molecular connection of IL-32 with host defense mechanisms in TB (78). In another example, modular analysis was used to compare blood signatures of ATB, sarcoidosis and NoTB controls (43). Pathway and GO enrichment analysis have also been instrumental to the identification of type I IFN as key major signature of ATB in many studies (36, 42, 49, 55, 79).

Finally, aforementioned, it is extremely important to distinguish primary vs. secondary effects in mechanistic studies, in order to identify the key upstream regulators that will represent the best candidates to use in the clinic. A way to address this is to consider gene expression signatures as a network of disease associated dysregulations rather than a simple list of genes. There are many algorithms that have been developed for network analysis of gene expression data (also called gene network inference), including RN (80), ARACNE (81), or C3Net (82). Regardless of the algorithm used, gene network analysis aims to infer the physical and/or biological interactions between genes in a given system (e.g., cell type or disease state) based on the transcriptomic data derived from this system. The derived networks can then be further refined by comparison with biological interactions databases. For instance, a genome-wide analysis of more than 100 samples from cancerous and non-cancerous prostate tissue samples predicted 18,583 gene-gene interactions, of which 54 were previously validated as direct physical interactions in the literature, significantly narrowing down the set of genes as potential targets for intervention (83). Systems biology studies (which encompass also proteomic and/or metabolomics data collection) can also be extremely useful to improve the accuracy of the molecular networks. For instance, a recent study combined plasma metabolite and cytokine concentration with whole blood gene expression to effectively depict molecular dysregulations occurring in TB-diabetes co-affected patients that were not apparent at the transcriptomic level only (27). Promising targets derived from the network analysis can then be further assessed for biological function *in vitro* using genetic interference or *in vivo* using knockout animal models.

## Future Perspectives

While there is still a long way to tackle all the obstacles associated with the control and eradication of TB, clearly host transcriptomics provide an invaluable tool to advance toward this goal. Host transcriptomic studies have not only identified novel targets for diagnostic and prognostic tests, but also improved our knowledge on TB-specific immune mechanisms. At the same time, these studies have highlighted issues such as the need to take in consideration similar pathologies and co-infection status in disease cohorts, the importance to compare whole blood vs. cell subsets and other tissues, and the versatility of bioinformatics tools available for the identification of gene signatures.

We have recently identified universal guidelines to consider when using the transcriptomic tool kit for the identification of diagnostic or mechanistic gene signatures (18). Here, by analyzing and compiling previously published TB transcriptomic studies, we further developed these guidelines for the optimal design and analysis of future transcriptomic studies to tackle the outstanding needs in the TB field. Future diagnostic studies should aim to identify disease signatures of very few genes, but with high sensitivity across all human populations affected, and high specificity against similar pathologies to TB, such as sarcoidosis. Additionally, there is a need to improve the accuracy of current infection state definitions (and associated diagnostic tests) that better reflect the TB disease spectrum. Additional work on prospective cohorts of LTBI progression to ATB is needed to improve the diagnosis efficacy of infected individuals at risk of developing active disease. The ultimate goal of mechanistic studies is to gather knowledge about cell types and gene networks that are relevant in a given disease. This could serve to determine dysregulated genes, paving the way for the identification of novel therapeutic procedures.

Finally, all studies should ensure their data is made publicly available, along with all required annotations for interpretation, to facilitate re-use by the research community (84). Such metadata analyses have already shown promising success in identifying both diagnostic and mechanistic signatures of TB. By re-analyzing all publicly available blood TB transcriptomics datasets together, Blankley et al. identified five genes that were consistently differentially expressed in active TB compared to LTBI or NoTB across all datasets, and thus represent promising candidates for blood based mRNA TB diagnosis (79). Using a similar approach, Sweeney et al identified a 3-gene set that distinguishes active TB from LTBI or other diseases, and validated its discriminatory power across 14 datasets with a total of more than 2,500 samples (75). Combining modular and gene-set enrichment analysis on publicly available data from 8 studies of whole blood/PBMC transcriptomics, Joosten et al. highlighted the importance of TREM1 signaling in active TB over the more typically reported type I IFN signaling (85). Thus, metadata analyses can provide robust diagnostic gene candidates and guide the choice of biological processes to focus on for future mechanistic studies.

## Author Contributions

JB, MB, MP, and CL wrote the manuscript. All authors participated to the literature review and edited the manuscript.

### Conflict of Interest Statement

The authors declare that the research was conducted in the absence of any commercial or financial relationships that could be construed as a potential conflict of interest.
